# Myelination induces axonal hotspots of synaptic vesicle fusion that promote sheath growth

**DOI:** 10.1016/j.cub.2021.06.036

**Published:** 2021-09-13

**Authors:** Rafael G. Almeida, Jill M. Williamson, Megan E. Madden, Jason J. Early, Matthew G. Voas, William S. Talbot, Isaac H. Bianco, David A. Lyons

**Affiliations:** 1 Centre for Discovery Brain Sciences, University of Edinburgh, Edinburgh, UK; 2 Department of Developmental Biology, Stanford University, Stanford, CA, USA; 3 National Cancer Institute, Frederick, MD, USA; 4 Department of Neuroscience, Physiology and Pharmacology, UCL, London, UK

**Keywords:** neuronal activity, synaptic vesicle fusion, axon, myelination, zebrafish, oligodendrocyte

## Abstract

Myelination of axons by oligodendrocytes enables fast saltatory conduction. Oligodendrocytes are responsive to neuronal activity, which has been shown to induce changes to myelin sheaths, potentially to optimize conduction and neural circuit function. However, the cellular bases of activity-regulated myelination *in vivo* are unclear, partly due to the difficulty of analyzing individual myelinated axons over time. Activity-regulated myelination occurs in specific neuronal subtypes and can be mediated by synaptic vesicle fusion, but several questions remain: it is unclear whether vesicular fusion occurs stochastically along axons or in discrete hotspots during myelination and whether vesicular fusion regulates myelin targeting, formation, and/or growth. It is also unclear why some neurons, but not others, exhibit activity-regulated myelination. Here, we imaged synaptic vesicle fusion in individual neurons in living zebrafish and documented robust vesicular fusion along axons during myelination. Surprisingly, we found that axonal vesicular fusion increased upon and required myelination. We found that axonal vesicular fusion was enriched in hotspots, namely the heminodal non-myelinated domains into which sheaths grew. Blocking vesicular fusion reduced the stable formation and growth of myelin sheaths, and chemogenetically stimulating neuronal activity promoted sheath growth. Finally, we observed high levels of axonal vesicular fusion only in neuronal subtypes that exhibit activity-regulated myelination. Our results identify a novel “feedforward” mechanism whereby the process of myelination promotes the neuronal activity-regulated signal, vesicular fusion that, in turn, consolidates sheath growth along specific axons selected for myelination.

## Highlights

• Live-imaged synaptic vesicular fusion during myelination of zebrafish spinal axons
• Neuron-subtype specific differences in axonal vesicular fusion
• Onset of myelination coincides with and promotes axonal vesicular fusion
• Activity and vesicular fusion promote sheath stability and growth

## Graphical abstract

**Figure:**
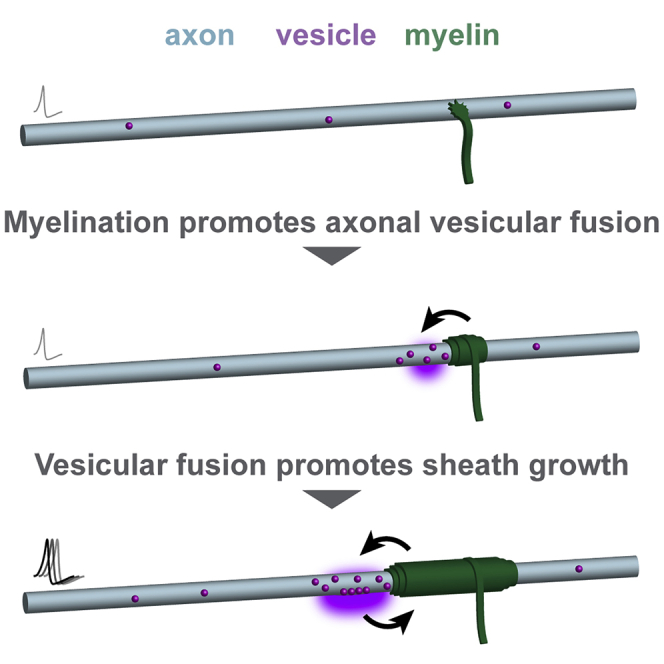

## Teaser

Almeida et al. show that fusion of synaptic vesicles along axons in the developing zebrafish spinal cord is as frequent as at presynaptic terminals in some neuronal subtypes. In these neurons, axonal vesicular fusion is promoted by myelination and in turn consolidates sheath formation and growth.

## Introduction

Ensheathment of axons by myelin drastically changes their conduction properties, enabling fast saltatory conduction of action potentials^1^ and providing axons with metabolic support.^2^, ^3^, ^4^ Dynamic changes to axonal myelination occur throughout life^5^ (e.g., through the differentiation of oligodendrocytes that form new myelin sheaths along previously unmyelinated or partially myelinated axons) or through remodeling of existing myelin sheaths.^6^^,^^7^ Oligodendrocytes express numerous neurotransmitters receptors and are responsive to neuronal activity.^8^, ^9^, ^10^ Furthermore, optogenetic or chemogenetic stimulation of neuronal firing can promote myelination along manipulated axons *in vivo*.^11^^,^^12^ Indeed, experience-dependent changes in myelination induced by neuronal activity are increasingly implicated in numerous aspects of nervous system formation, function, and health.^13^, ^14^, ^15^, ^16^ However, the cellular bases for how neuronal activity along axons might regulate myelination *in vivo* are unclear. Recent studies suggested that the effects of neuronal activity on myelination are mediated by the release of synaptic vesicles,^17^^,^^18^ including in a neuron-subtype-specific manner.^19^^,^^20^ For example, in zebrafish, blocking vesicular fusion from specific neurons reduced myelin sheath number and length along their axons.^18^^,^^20^ Furthermore, live-imaging studies revealed that synaptic vesicles can accumulate along axons during myelination,^18^^,^^21^^,^^22^ leading to the model that vesicular cargo directly drives myelin sheath formation and/or growth.^23^ However, it is unclear whether synaptic vesicle fusion along the axon precedes and biases myelin targeting to more active axons or only consolidates myelin sheaths after their formation. It is also unclear whether vesicular fusion occurs stochastically along myelinated axons or whether there are discrete hotspots of vesicular fusion, and if so, how those are established. Furthermore, the bases for neuronal-subtype-specific differences in activity-regulated myelination *in vivo* are unclear.

To address these questions, we investigated the relationship between vesicular fusion and the myelination of individual axons *in vivo* over time, through a high-resolution live-imaging approach in living zebrafish. We found, surprisingly, that localized axonal synaptic vesicle fusion is promoted by myelination. This myelin-induced axonal vesicle fusion was enriched along non-myelinated heminodal domains, into which nascent sheaths grew. Blocking axonal vesicular fusion reduced the growth and in turn stable formation of nascent sheaths. Furthermore, enhancing neuronal activity and vesicular fusion accelerated the growth of stable sheaths. We also determined that different neuronal subtypes exhibited distinct levels of axonal vesicular fusion in line with whether they exhibit activity-regulated myelination.

Our results identify a novel feedforward mechanism whereby the process of myelination promotes the neuronal activity-regulated signal, vesicular fusion that, in turn, stimulates sheath formation and growth along specific axons selected for myelination.

## Results

### Synaptic vesicle fusion occurs along reticulospinal axons

**Figure 1:**
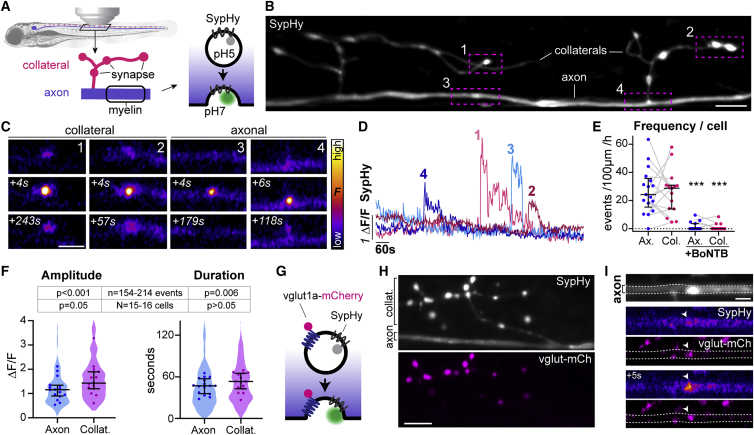
SypHy reveals vesicular fusion along reticulospinal axons (A) Morphology of reticulospinal axons in the developing zebrafish spinal cord. SypHy, a synaptophysin-pHluorin fusion protein, reports synaptic vesicle exocytosis. (B) Individual SypHy^+^ reticulospinal axon (dorsal up) with synapse-bearing collateral branches. (C and D) SypHy events at collaterals and axon (C) and fluorescence time courses (D). (E) SypHy frequency is similar in axons and collaterals (gray lines shows respective events of same cell); and abolished in BoNTB^+^ neurons (p = 0.82 Ax versus Col; p < 0.001 Ax versus Ax+BoNTB; p < 0.001 Col versus Col+BoNTB; Mann-Whitney test, 17 control axons from 16 animals; 15 BoNTB axons from 15 animals). (F) Amplitude and duration of SypHy events in reticulospinal axons. Violin plots represent all analyzed events and circles denote average per axon (p = 0.054 Ax versus Col amplitude, p = 0.264 Ax versus Col duration, Mann-Whitney tests; n = 154 axonal and 214 collateral events from N = 16 axons from 15 animals). (G–I) vglut1a-mCherry co-expression with SypHy (G) shows co-localization at synaptic terminals in collateral branches (H) and at axonal puncta (I). Scale bars, 5 μm (B, H, and I), 2 μm (C). Graphs display median and interquartile range. See also Figure S1 and Videos S1, S2, and S3.

To elucidate the mechanisms by which synaptic vesicle fusion and neuronal activity regulate myelination in intact neural circuits, we first aimed to define when and where synaptic vesicles fuse along axons. To do so, we performed live-cell imaging in developing zebrafish, which enable optical and genetic access to single neurons in an intact nervous system over time. We first characterized synaptic vesicular fusion along individual reticulospinal axons in the spinal cord, which become progressively myelinated in an activity/synaptic vesicle fusion-dependent manner from 3 days post-fertilization (dpf) onward.^20^ To do so, we used SypHy,^24^ in which synaptophysin, a transmembrane protein specifically localized to synaptic vesicles,^25^, ^26^, ^27^ is fused to four pH-sensitive pHluorin molecules. The pHluorin molecules are targeted to an intralumenal loop of synaptophysin and face the acidic vesicle lumen, where their fluorescence is quenched. SypHy only fluoresces when the pH is neutralized upon synaptic vesicle exocytosis (Figure 1A). The high signal-to-noise ratio provided by the multiple pHluorin molecules of this SypHy reporter enabled *in vivo* imaging of vesicular fusion. As previously reported,^28^^,^^29^ a fraction of synaptophysin molecules reside on the plasma membrane, reflected by residual SypHy fluorescence that revealed individual reticulospinal axon morphology (Figure 1B). Reticulospinal neurons have a large principal axonal shaft, which projects from the brain through the spinal cord and is myelinated over time, as well as regularly spaced collateral branches that remain unmyelinated and contain presynaptic terminals.^30^ 1-Hz imaging of reticulospinal neurons at 4–5 dpf, during active myelination, revealed bright, focal fluorescence increases at collateral branches with presynaptic terminals, as expected and also along the axon shaft (hereafter designated “axonal” events) (Figures 1C and 1D; Video S1). Although SypHy event frequency was variable between individual neurons, axonal and collateral SypHy frequencies were, on average, similar in the reticulospinal neuron population (Figure 1E). Individual events were also comparable in amplitude and duration but had slightly higher amplitude and duration in collateral branches (Figure 1F). To validate SypHy as reporting vesicular fusion, we co-expressed mCherry-tagged botulinum toxin B (BoNT-B),^31^ which cleaves synaptobrevin-2, a vesicular transmembrane protein essential for exocytosis.^32^ Both axonal and collateral SypHy activity were blocked by BoNTB-mCherry co-expression (Figures 1E and S1; Video S2) validating events at both locations as bona-fide vesicular fusion. Most SypHy events (>75%) along control axons occurred in discrete locations with no subsequent displacement of fluorescent puncta and are thus likely to represent sites of focal vesicular fusion (Figure S1; Video S3). A minority showed some displacement and also appeared insensitive to BoNT-B (Figure S1), which suggests they represent other vesicle populations or vesicular trafficking, and so were not considered further. To further validate SypHy as reporting the fusion of neurotransmitter-filled synaptic vesicles, we co-expressed mCherry-tagged glutamate transporter vglut1a (Figures 1G–1I), because reticulospinal axons are glutamatergic. We observed that vglut1a-mCherry and SypHy were co-localized in all collateral branches (Figure 1H), and mCherry^+^ puncta were also present along axons where they co-localized with SypHy events (Figures 1I and S1).

Video S1. SypHy activity along a single control reticulospinal axon, related to Figure 1 Raw fluorescence (top) and ΔF/F (bottom) in a SypHy-expressing control reticulospinal axon at 4 days postfertilization, when myelination (not shown here) is ongoing. Arrowheads point to examples of SypHy events occurring along collaterals (where presynaptic terminals are located) and the main axonal projection. Time indicated in min:sec.

Video S2. SypHy activity along a single BoNT-B silenced reticulospinal axon, related to Figure 1 Raw fluorescence (top) and ΔF/F (bottom) in a SypHy-expressing BoNTB-mCherry co-expressing reticulospinal axon at 4 days postfertilization, when myelination (not shown here) is ongoing. Arrowheads point to examples of SypHy events occurring along collaterals (where presynaptic terminals are located) and the main axonal projection. Note silencing of SypHy activity in BoNTB axon. Time indicated in min:sec.

Video S3. SypHy events are mostly static, related to Figure 1 Raw fluorescence (top) and ΔF/F (bottom) of three further examples of wildtype larva in which individual axons express SypHy. Note that SypHy events can be static (white arrowheads) or show displacement after their appearance (yellow arrowheads), quantified in Figure S1. Time indicated in min:sec.

Collectively, our data reveal that *in vivo*, synaptic vesicles fuse not only at presynaptic terminals, as expected, but also along the length of reticulospinal axons during stages in which they become myelinated.

### Axonal vesicular fusion emerges with and is stimulated by myelination

**Figure 2:**
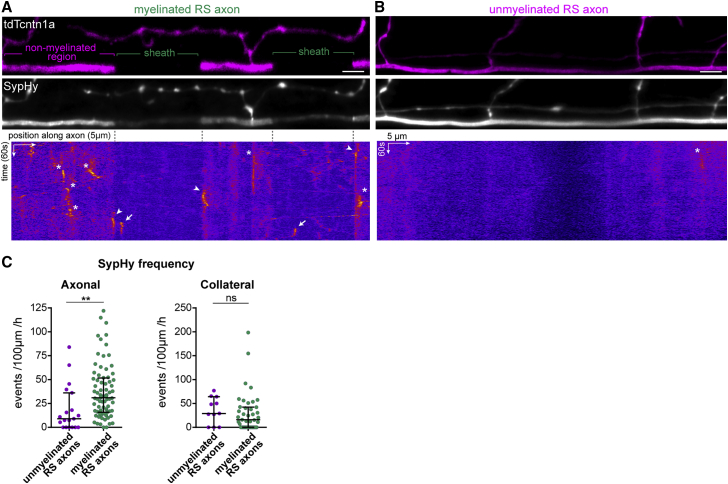
Axonal vesicular fusion coincides with the onset of myelination (A and B) tdTcntn1a profile of a myelinated (A) and a not-yet myelinated (B) reticulospinal axon. tdTcntn1a is excluded from myelinated regions along axons. SypHy average projections and corresponding kymographs of the axonal segments in panels below. Basal SypHy signal is decreased along sheaths, suggesting surface SypHy displacement by myelin. SypHy events are present in non-myelinated regions (asterisks), myelinated regions (arrows) and at the tdTcntn1a^+^/tdTcntn1a^−^ border (arrowheads). (C) Axonal, but not collateral, SypHy frequency in myelinated reticulospinal axons is greater than in unmyelinated axons (axonal, p = 0.001; collateral, p = 0.30; Mann-Whitney test; axonal rates, 19 unmyelinated axons from 19 animals and 75 myelinated axons from 68 animals). Scale bars, 5 μm, 60 s (A and B). Graphs display median and interquartile range. See also Figure S2 and Video S4.

To directly examine the relationship between axonal vesicular fusion and the myelination of individual axons, we combined SypHy together with tdtomato-contactin1a (tdTcntn1a), our axonal reporter of myelination that is excluded from areas of the axon ensheathed by myelin.^20^^,^^33^ We readily detected SypHy events along reticulospinal axons undergoing myelination, both in myelinated (tdTcntn1a^−^) and non-myelinated (tdTcntn1a^+^) regions (Figure 2A; Video S4). To examine the relationship between axonal vesicular fusion and the onset of myelination in more detail, we focused on reticulospinal axons that were not yet myelinated at 4 dpf (Figure 2B). We found axonal SypHy activity was less frequent in axons not yet undergoing myelination than in axons undergoing myelination (Figure 2C). In contrast, SypHy event frequency at collaterals was comparable to that of myelinated axons (Figure 2C). The observation that the onset of myelination coincides with a specific increase in axonal but not collateral vesicular fusion suggests that myelination itself might in fact stimulate vesicular fusion from axons.

Video S4. Relationship between SypHy activity and myelination, related to Figure 2 An individual reticulospinal axon co-expressing the tdTomato-cntn1a reporter of myelination, whose gaps indicate myelin sheaths, and SypHy reporter, enabling accurate quantification of SypHy activity along myelinated and unmyelinated regions. Arrowheads point to examples of SypHy events occurring along collaterals and axon. Time indicated in min:sec.

**Figure 3:**
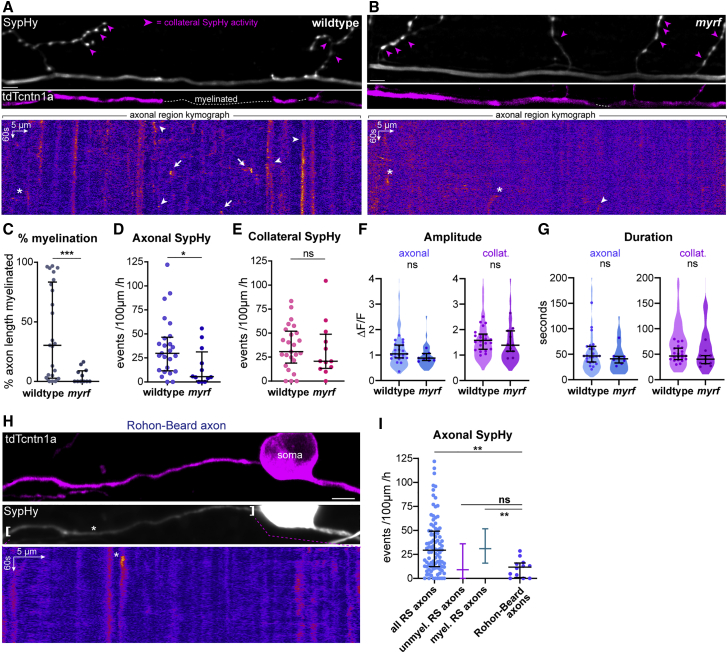
Myelination promotes vesicular fusion along reticulospinal axons (A and B) SypHy activity and myelination profile of reticulospinal axons in wild-type (WT) (A) and hypomyelinated *myrf*^UE70^ mutants (B). Magenta arrowheads indicate collateral SypHy activity. Kymographs represent SypHy activity of the axonal region above. (C) Myelin coverage in WT and *myrf* (p = 0.002, Mann-Whitney test, 25 axons from 19 WT animals and 12 axons from 11 *myrf* animals). (D and E) Axonal (D), but not collateral (E), SypHy frequency is decreased in *myrf* mutants (D, p = 0.022; E, p = 0.476; Mann-Whitney test, N as in C). (F and G) Amplitude (F) and duration (G) of SypHy events is similar in WT and *myrf* mutants. Violin plots represent all analyzed events and circles denote average per axon (WT versus myrf; F, p = 0.112 ax, p = 0.396 col; G, p = 0.317 ax, p = 0.144 col; Mann-Whitney tests, n = 39–216 axonal and 77–181 collateral events from N = 23 axons from 17 WT animals and 11 axons from 10 *myrf* animals). Note that axonal versus collateral comparisons are significant: (F) p < 0.001 WT, p = 0.008 *myrf*, ANOVA with Bonferroni correction for multiple comparisons; (G) p = p < 0.001 WT, p = 0.088 *myrf*, Kruskal-Wallis with Dunn’s multiple comparisons test (for all, N = 23 WT axons and 11 myrf axons). (H) Myelination profile and SypHy activity of a Rohon-Beard axon. Kymograph of bracketed region. (I) Axonal SypHy frequency in rarely myelinated Rohon-Beard axons is significantly reduced compared to reticulospinal axons (p = 0.006 all RS versus Rohon-Beard, p > 0.05 unmyelinated. RS versus Rohon Beard, p = 0.001 myelinated. RS versus Rohon Beard; Kruskal-Wallis with Dunn’s multiple comparison test, 94 RS axons in 87 animals and 12 Rohon-Beard axons from 11 animals). Scale bars, 5 μm, 60 s (A, B, and H). Graphs display median and interquartile range. See also Videos S5 and S6.

To test this directly, we sought to specifically manipulate myelination. We reduced CNS myelin formation by genetically disrupting zebrafish *myrf*, which encodes an oligodendrocyte-specific transcription factor required for myelin production.^34^, ^35^, ^36^ We recently generated the mutant allele *myrf*^UE70^, containing a frameshift mutation in the second *myrf* exon that introduces a premature stop codon. This mutation disrupts oligodendrocyte development and leads to severe hypomyelination in the CNS.^37^ We confirmed that individual reticulospinal axons were hypomyelinated along their length in *myrf* mutants (Figures 3A–3C). At 4–5 dpf, most reticulospinal axons in wild-type siblings were in the process of being myelinated (median 30% axonal length myelinated, 25 axons); whereas *myrf* mutant siblings had little to no myelin (median of 0% axonal length myelinated, 12 axons) (Figure 3C). When we imaged SypHy, we found that the frequency of axonal SypHy events was decreased in *myrf* mutants, whereas collateral SypHy event frequency was not significantly affected (Figures 3D and 3E; Video S5). In contrast to event frequency, the amplitude and duration of SypHy events were similar between wild-type and mutants (Figures 3F and 3G). To further address the relationship between myelination and axonal vesicular fusion, we also imaged SypHy in axons fated to remain unmyelinated. Rohon-Beard neurons in the dorsal spinal cord project large axons accessible to oligodendrocyte processes^33^ but that are essentially unmyelinated.^20^^,^^38^ The frequency of SypHy events along Rohon-Beard axons was significantly lower than in reticulospinal neurons as a whole and comparable to not-yet myelinated reticulospinal axons (Figures 3H and 3I; Video S6). Taken together, these data indicate that, surprisingly, myelination itself promotes vesicular fusion along myelinated axons, revealing a novel role for myelin in regulating axonal physiology.

Video S5. SypHy activity along an individual reticulospinal axon in a wildtype larva and in an hypomyelinated *myrf* mutant larva, related to Figure 3 These larva are an homozygous wildtype sibling and a homozygous mutant sibling. Note reduction in axonal, but not collateral SypHy events in mutant. Time indicated in min:sec.

Video S6. SypHy activity in a Rohon-Beard neuron, related to Figure 3 Raw fluorescence of an individual Rohon-Beard axon expressing SypHy. Scalebar 5μm, time indicated in min:sec.

### Axonal vesicular fusion occurs in heminodal hotspots into which sheaths grow

**Figure 4:**
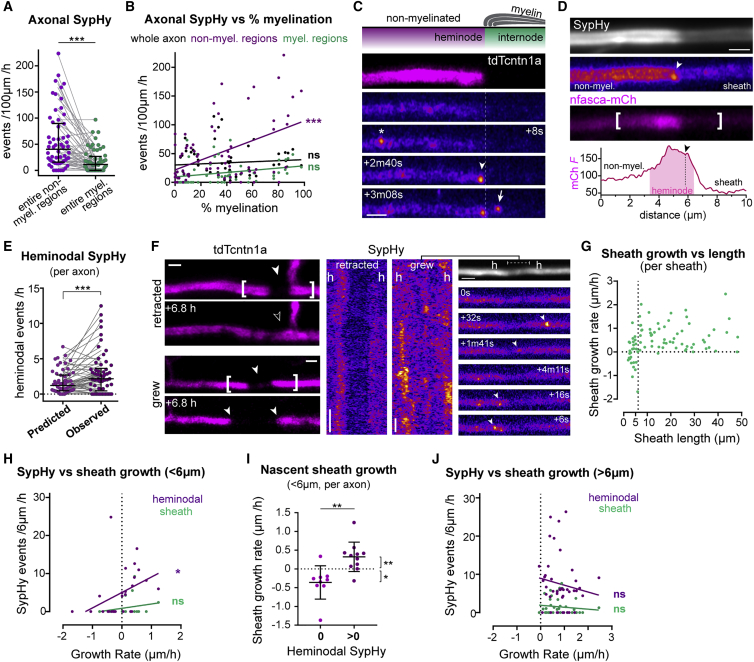
Axonal vesicular fusion is enriched at heminodes during nascent sheath growth (A) Within myelinated axons, SypHy is more frequent in non-myelinated regions (p < 0.0001, Wilcoxon matched-pairs signed-rank test, 58 myelinated axons from 53 animals). (B) Percent myelination correlates with SypHy frequency in non-myelinated regions (Pearson’s r, 0.46, p < 0.0001; in myelinated regions r, 0.25, p = 0.05; in whole axon r, 0.10, p = 0.43). (C) SypHy activity near a putative heminode, annotations as in Figure 2A. (D) nfasca-mCherry is enriched at putative heminodes (arrowhead, heminodal SypHy event). Graph indicates mCherry fluorescence intensity profile (in a.u.) along bracketed region. (E) Observed heminodal Syphy frequency is higher than predicted if events in non-myelinated regions were uniformly distributed (p = 0.0004, Wilcoxon matched-pairs rank test, 58 axons from 53 animals). (F) tdTcntn1a profiles and SypHy activity in retracting and growing nascent sheaths (h = heminode). (G) Relation between sheath length and growth rate, note sheaths <6 μm may grow or shrink, but >6 μm sheaths mostly grow (85 sheaths from 39 axons in 35 fish). (H) Nascent sheath growth rate correlates with heminodal SypHy frequency (Pearson’s r: 0.39, p = 0.043) but not under the sheath (Pearson’s r: 0.31, p = 0.111). 85 sheaths from 39 axons from 36 animals. (I) Nascent sheaths with heminodal SypHy activity grow faster than those without (p = 0.001 with versus without, Mann-Whitney test; p = 0.01 and p = 0.039 non-zero growth rate with and without heminodal SypHy, one-sample Wilcoxon test; 23 sheaths in N = 14 axons from 13 animals). (J) Growth rate of sheaths >6 μm does not correlate with SypHy activity (heminodal Pearson’s r: −0.14, p = 0.381; sheath Pearson’s r: −0.12, p = 0.441). Scale bars, 2 μm (C, D, and F), 60 s (F). Graphs display median and interquartile range. See also Figure S2.

We previously showed that blocking vesicular fusion with tetanus toxin along individual reticulospinal axons reduced the number and length of their myelin sheaths.^20^ Recent studies have proposed that synaptic vesicles accumulate and fuse under myelin sheaths, releasing signals that promote myelin growth.^21^^,^^22^ To address precisely where and when axonal vesicular fusion occurs, we examined the subcellular distribution of SypHy events along single myelinated axons in more detail. Unexpectedly, we observed an over 3-fold higher frequency of SypHy events in non-myelinated regions of axons undergoing myelination compared to their myelinated regions (Figure 4A). Furthermore, in 70 myelinated axons from 57 animals, we found that SypHy activity in non-myelinated regions correlated positively with the extent of an axon’s myelination (Figure 4B), such that axons with more SypHy events in their non-myelinated regions were also the axons with more myelin coverage along their length. In contrast, SypHy activity in myelinated regions did not correlate with the extent of an axon’s myelination (Figure 4B). Our data suggest that significant vesicular fusion occurs at non-myelinated sites where myelin sheaths grow into, after the myelinating process becomes targeted to the axon.

Exploiting the sub-cellular resolution afforded to us by our reporters, we next observed that within non-myelinated regions, many events were localized adjacent to the ends of myelin sheaths (within 3 μm) (Figure 4C, arrowhead). In axons undergoing myelination, these hotspots immediately adjacent to myelin—into which growing sheaths are likely to grow—are called heminodes. Heminodes often display an enriched localization of proteins that ultimately cluster at nodes of Ranvier as neighboring sheaths grow toward one another.^39^, ^40^, ^41^ Co-expression of SypHy and nfasca-mCherry, a nodal cell adhesion molecule, showed that non-myelinated regions adjacent to the ends of myelin sheaths could indeed be enriched in Nfasca during the myelination of zebrafish reticulospinal axons (Figure 4D), confirming that they had heminodal characteristics. Although this analysis revealed that SypHy events could localize to heminodal regions, it also revealed that they were independent from nfasca-mCherry vesicle trafficking dynamics, suggesting that they are distinct vesicle populations (Figure S2). Quantitative analyses of their subcellular localization revealed that heminodal SypHy events occurred at a frequency higher than expected if they were uniformly distributed along the entire non-myelinated regions (Figure 4E). Together, these data indicate that axonal vesicular fusion does not occur stochastically along myelinated axons but rather is increased following myelination at heminodal hotspots, into which nascent myelin sheaths grow.

### Axonal vesicular fusion promotes and consolidates nascent sheath growth

The enrichment of SypHy events at heminodal hotspots into which sheaths grow suggested that localized vesicular fusion might promote sheath growth into those regions. To relate axonal vesicular fusion and the growth rate of individual sheaths, we imaged 85 myelin sheaths in 39 individual axons before and 2–8 h after SypHy imaging at 4–5 dpf (Figure 4F). When we plotted the growth rate of individual sheaths against their initial length, we noticed that the 28 nascent sheaths shorter than 6 μm were equally likely to grow in length (14/28, 50%) or shrink (14/28, 50%). In contrast, the 57 sheaths longer than 6 μm rarely shrank (1/57, 2%) (Figure 4G). This length-dependent fate suggests that newly formed sheaths are vulnerable to retraction but that factors promoting their elongation past a 6-μm threshold ensure their stability. Indeed, all complete retractions were of sheaths shorter than 6 μm. We examined SypHy activity in more detail in the vicinity of nascent sheaths shorter than 6 μm (Figures 4F–4I). Overall, heminodal SypHy event frequency correlated positively with the speed of nascent sheath growth, whereas SypHy activity under nascent sheaths did not (Figure 4H). Remarkably, 13/15 (87%) nascent sheaths with heminodal SypHy activity grew in length, compared to only 3/13 (23%) of sheaths with zero SypHy activity. Furthermore, on average, nascent sheaths with heminodal SypHy activity had a growth rate significantly above zero (i.e., grew in length), whereas nascent sheaths with no associated heminodal SypHy activity had a growth rate significantly below zero (i.e., shrank) (Figure 4I). In contrast, there was no correlation between SypHy and growth of stabilized sheaths (>6 μm) over this short timescale (Figure 4J).

**Figure 5:**
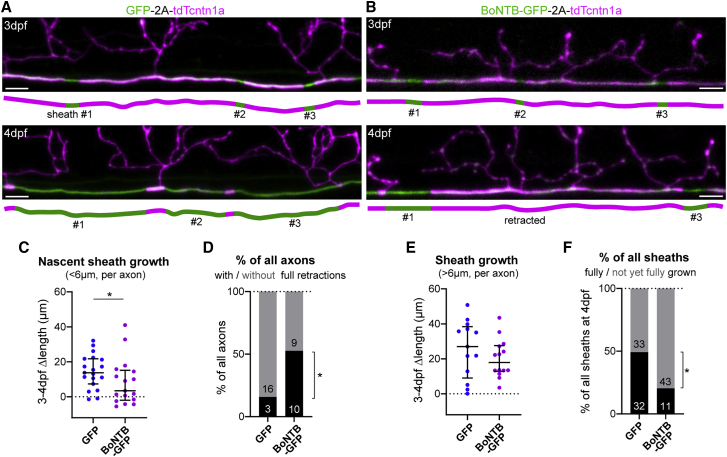
Axonal vesicular fusion consolidates nascent myelin sheaths (A and B) Examples of 3–4 dpf time-course analyses of myelination of control RS axons (GFP-2A-tdTcntn1a) and RS axons with blocked vesicular fusion (BoNTB-GFP-2A-tdTcntn1a). (C) Nascent sheaths in axons with blocked vesicular fusion grow slower (p = 0.048, Mann-Whitney test, 19 control axons from 18 animals and 17 BoNTB axons from 15 animals). (D) Proportion of axons with and without full sheath retractions (p = 0.038, Fisher’s exact test). Numbers in bars indicate absolute number of axons. (E) Sheaths >6 μm have a non-significant slower median growth in BoNTB-silenced axons (p = 0.488, Mann-Whitney test, 13 control axons from 13 animals and 14 BoNTB axons from 13 animals). (F) Proportion of sheaths at 4 dpf fully grown (flanked by nodes and/or collaterals) or still with space to grow (p = 0.001, Fisher’s exact test). Numbers in bars indicate absolute number of sheaths. Scale bars, 5 μm (A and B). Graphs display median and interquartile range. See also Figure S2.

Thus, our data show vesicular fusion adjacent to sites of sheath formation positively correlates with faster sheath growth, suggesting it promotes early myelin growth and stabilizes nascent sheaths. To test how vesicular fusion affects early myelination, we expressed BoNTB-EGFP in individual reticulospinal neurons to disrupt axonal vesicular fusion, and tdTcntn1a to assess their myelination profile between 3 and 4 dpf (Figures 5A and 5B). We found that nascent sheaths (shorter than 6 μm) in control axons grew significantly more than nascent sheaths in BoNTB-silenced axons (Figure 5C). Furthermore, full retractions of nascent sheaths occurred in a larger proportion of BoNTB-B silenced axons compared to control axons (Figure 5D). These data are consistent with our model that vesicular fusion promotes the growth and thus stable formation of nascent myelin sheaths.

### Neuronal activity stimulates vesicle fusion and sheath growth

**Figure 6:**
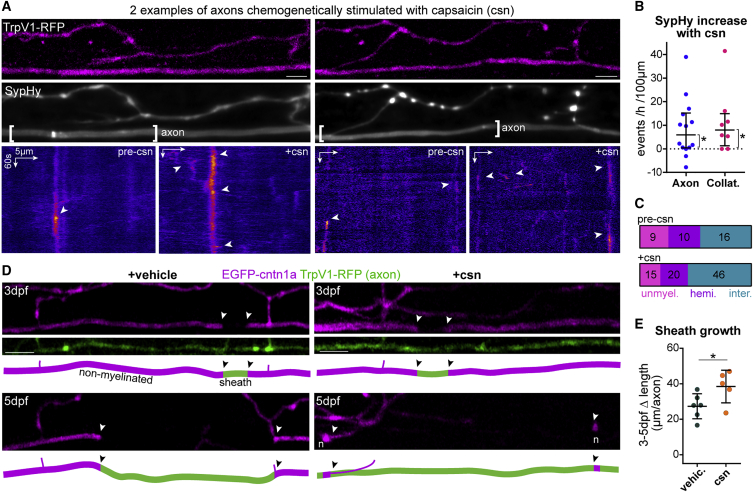
Chemogenetic stimulation of vesicular fusion promotes sheath growth (A) Two examples of SypHy activity in TrpV1-tagRFP^+^ reticulospinal axons before (pre-csn) and during capsaicin treatment (+csn). (B) Capsaicin increased axonal and collateral SypHy in TrpV1^+^ neurons above baseline (difference from zero: p = 0.013 axonal, p = 0.031 collateral, Wilcoxon signed-rank test, 14 axons and 8 collaterals from 14 and 8 animals, respectively). (C) Axonal event distribution before (n = 35 events) and after (n = 81 events) csn treatment. (D) Single TrpV1^+^ axons treated with vehicle or capsaicin throughout myelination (arrowheads, putative heminodes; n, putative nodes of Ranvier). (E) Stimulation promotes sheath growth (3–5 dpf change: p = 0.04, Student’s t test). 6 control axons and 5 stimulated axons from 6 and 5 animals, respectively. Scale bars, 5 μm (A), 10 μm (D), 60 s (A). Graphs in (B) shows median and interquartile range and graphs in (E) display mean and standard deviation. See also Figure S3 and Videos S7 and S8.

We next wanted to test whether vesicular fusion could also regulate the growth of stabilized sheaths. We observed that sheaths longer than 6 μm at 3 dpf showed a trend toward reduced growth in BoNTB-silenced axons (Figure 5E), and at 4 dpf there was a significant reduction of sheaths fully grown such that they are flanked by nodes and/or collaterals (Figure 5F). Given these data, we wanted to investigate whether increasing the activity of individual reticulospinal axons can promote sheath growth. To enhance vesicular fusion along single reticulospinal axons, we expressed tagRFP-tagged rat TRPV1, a cation channel specifically activated by capsaicin, which drives neuronal excitation and activity-dependent vesicular fusion and has been used in zebrafish to increase the firing frequency of spinal neurons.^42^ In individual TRPV1-RFP^+^ axons, application of 1 μM capsaicin (but not vehicle) increased intra-axonal calcium activity within 15 min, assessed with axon-tethered GCaMP7s,^43^ compared to their baseline and to control axons (Figure S3; Video S7). This demonstrates a specific increase in neural activity in treated transgenic axons with our approach. Capsaicin also significantly increased the frequency of both axonal and synaptic SypHy events, with 10/12 TrpV1^+^ axons showing increased axonal SypHy (overall average 1.6-fold increase) (Figures 6A and 6B; Video S8). In those axons in which we could differentiate between non-myelinated and myelinated regions, we observed an increase in SypHy activity in all axonal regions following capsaicin stimulation (Figure 6C). These data indicate that SypHy-reported axonal vesicular fusion is activity-regulated, and is increased with our chemogenetic approach. We then took advantage of the temporal control that this inducible approach afforded us to increase vesicular fusion in individual axons specifically throughout their period of myelination, by treating larva with capsaicin every day for 4 h from 3 to 5 dpf. We imaged their myelination profile using EGFP-cntn1a (Figure 6D) and found that sheaths on stimulated axons grew significantly more; with sheaths in both conditions growing from 8 ± 4 μm at 3 dpf to 45 ± 6 μm by 5 dpf in stimulated axons, compared to 36 ± 5 μm in controls (Figure 6E). Thus, stimulating activity specifically promotes an increase in myelin sheath growth, even beyond the early stages of consolidation of nascent sheaths. Because vesicular fusion along axons is itself stimulated by the onset of myelination (Figures 2 and 3), our data reveal a “feedforward” regulation by which myelination promotes the activity-regulated mechanism that consolidates and enhances further myelination of individual axons in developing neural circuits.

Video S7. Ca^2+^ response to capsaicin (csn) stimulation in a TrpV1^+^ expressing axon, related to Figure 3 Raw fluorescence of an individual reticulospinal axon co-expressing TrpV1-tagRFP and axon-GCaMP7s before (pre-csn) and during (+csn) capsaicin treatment. Note increase in axonal calcium activity. Time indicated in min:sec.

Video S8. SypHy response to capsaicin (csn) stimulation in a TrpV1^+^ expressing axon, related to Figure 6 Raw fluorescence (top) and ΔF/F (bottom) of an individual reticulospinal axon co-expressing TrpV1-RFP and SypHy before (pre-csn) and during (+csn) capsaicin treatment. Note increase in SypHy activity. Time indicated in min:sec.

### Reduced axonal vesicular fusion in COPA axons

**Figure 7:**
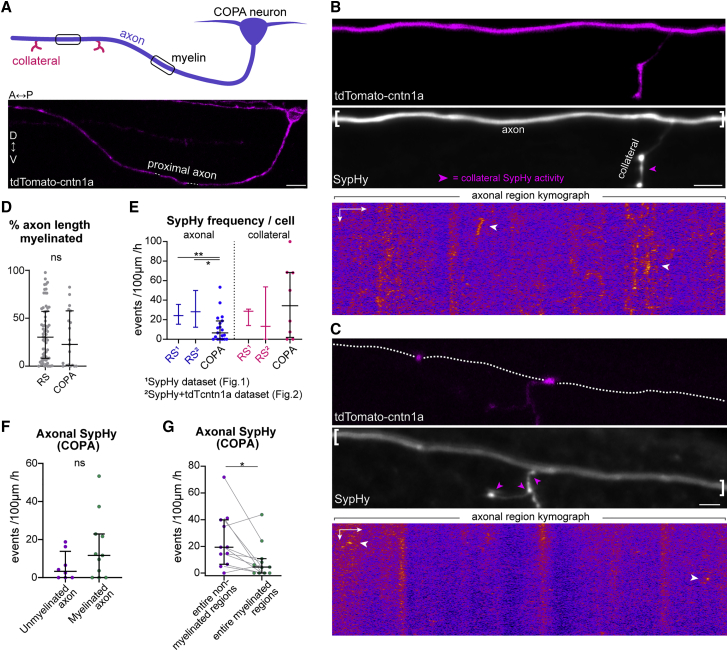
COPA neurons have limited axonal vesicular fusion (A) Morphology of COPA axons in the developing zebrafish spinal cord. (B and C) Two examples of COPA axons, their myelination profiles and SypHy activity. Magenta arrowheads indicate collateral SypHy activity. Kymographs represent SypHy activity of bracketed axonal region. (D) Myelin coverage is similar in reticulospinal and COPA axons (p = 0.291, Mann-Whitney test, 66 RS axons in 57 animals and 19 COPA axons from 19 animals). (E) Axonal, but not collateral SypHy frequency is reduced in COPA axons compared to reticulospinal datasets (axonal: p = 0.043 RS1 versus COPA, p = 0.002 RS2 versus COPA; collateral: p > 0.99 RS1 versus COPA, p > 0.99 RS2 versus COPA; Kruskal-Wallis with Dunn’s multiple comparison test; N as in Figures 1 and 2 and D). (F) Axonal SypHy frequency is comparable in myelinated and unmyelinated COPA axons (p = 0.237, Mann-Whitney test, 8 unmyelinated and 11 myelinated COPA axons from 19 animals). (G) Within myelinated COPA axons, SypHy frequency is higher in non-myelinated regions (p = 0.024, Wilcoxon matched-pairs signed-rank test, 11 myelinated COPA axons from 11 animals). Scale bars, 10 μm (A), 5 μm, and 60 s (B and C). Graphs display median and interquartile range. See also Figure S4 and Video S9.

We previously found that neuronal activity regulates myelination in a neuronal subtype-specific manner. Although we previously showed that COPA axons are of similar caliber and neurotransmitter phenotype to reticulospinal neurons and become myelinated at similar stages, we found that abrogating vesicular fusion does not reduce COPA myelination,^20^ and the basis for this difference to reticulospinal myelination remained unclear. To test whether differences in vesicular fusion properties underlie this distinction, we imaged SypHy and tdTcntn1a along COPA axons at 4–5 dpf (Figures 7A–7C and S4; Video S9). We first confirmed that at these stages COPA and reticulospinal axons have similar extents of myelination (Figure 7D). Remarkably, although we did not observe a difference in vesicular fusion along collateral branches, both unmyelinated and myelinated COPA axons had significantly reduced axonal vesicular fusion compared to reticulospinal axons (compare Figure 2C with 7E). These data indicate that the extent of vesicular fusion along axons can be a neuron-type-specific property. Interestingly, despite the reduced overall axonal SypHy frequency in COPA neurons, we still observed a trend toward more frequent SypHy activity in myelinated versus unmyelinated COPA axons (Figure 7F). We also observed significantly more frequent vesicular fusion in non-myelinated regions of COPA axons compared to myelinated regions (Figure 7G), as seen for reticulospinal axons. Together, these data suggest that the onset of myelination induces changes along both axonal types that facilitate localized vesicular fusion, and it is the levels of vesicular fusion that ultimately influence myelination.

Video S9. SypHy activity in a COPA axon, related to Figure 7 Raw fluorescence of an individual COPA axon expressing SypHy. Scalebar 5μm, time indicated in min:sec.

## Discussion

We and others have previously shown that vesicular fusion is required for appropriate myelination along axons.^18^^,^^20^ Here, we make the unexpected observation that the onset of myelination itself stimulates localized axonal vesicular fusion. In addition, we show that vesicular fusion is enriched in regions adjacent to myelin sheaths, and that vesicular fusion stimulates sheath growth. This is particularly important at the early stages following ensheathment, because it determines whether nascent sheaths are fully retracted from or stably maintained on axons. In addition, vesicular fusion also promoted elongation of stabilized sheaths. Furthermore, we found that the overall frequency of axonal vesicular fusion determines the impact that it has on myelination in a neuron subtype-specific manner.

Our data clarify how activity-regulated vesicular fusion affects myelination. We and others have previously shown that abrogation of vesicular release from neurons reduces sheath number and length,^18^^,^^20^ but it has remained unclear to what extent sheath formation and elongation are distinctly regulated by activity. Here, we propose that activity-regulated vesicular fusion generally promotes the growth of myelin sheaths. This has the consequence of consolidating the stable formation of nascent sheaths that are more prone to shrinking and retraction, as we showed before.^44^ The resolution afforded to us by our model enabled us to determine that once sheaths reach a length of ∼6 μm they only ever elongate, and our data support a continued role for vesicular fusion in promoting ongoing sheath elongation of stabilized, >6 μm sheaths.

As axons become fully myelinated along their length, vesicular fusion may continue to promote sheath stability, remodeling, or growth in thickness.^11^^,^^12^ Indeed, in 7 dpf animals, when reticulospinal axons are more fully myelinated along their lengths, axonal vesicular fusion remains at levels comparable to 4–5 dpf (Figure S2). Further analyses in mature circuits will determine the roles of this persistent axonal vesicular fusion.

The molecular mechanisms by which myelination promotes vesicular fusion, and by which vesicular fusion in turn promotes myelination, remain to be defined. For instance, does myelination locally concentrate vesicular fusion machinery in the underlying axon? Or does myelination drive other localized changes (e.g., to axonal diameter or the axonal cytoskeleton) that facilitate vesicular trafficking or fusion?^45^^,^^46^ Do similar mechanisms regulate the fusion of synaptic vesicles and other vesicles (e.g., those containing nodal components)^39^^,^^47^ along axons?

In turn, the cargo of vesicles that promote myelin growth warrants further investigation. Reticulospinal axons are glutamatergic,^48^^,^^49^ and many SypHy events co-localize with vesicular glutamate transporter vglut1a (Figure 1). Vesicular glutamate release has previously been implicated in myelination *in vivo*.^21^^,^^50^^,^^51^ It will be important to characterize the properties of glutamatergic release from multiple types of axon and to identify the oligodendrocyte receptors that mediate the effects observed in our study. Cargo of other vesicle types (e.g., dense-core vesicles) can also be released in an activity-dependent manner and have been implicated in myelination (e.g., BDNF);^50^^,^^52^^,^^53^ therefore, it is necessary to dissect the relative contribution of these diverse signals in regulating myelination in other anatomical regions, developmental stages, and even in the regeneration of myelin, which neuronal activity can also regulate.^14^^,^^54^, ^55^, ^56^

In summary, our *in vivo* imaging of synaptic vesicle fusion in axons undergoing myelination has revealed an unexpected feedforward model whereby the onset of myelination promotes localized axonal vesicular fusion that in turn promotes myelin growth. This may serve to ensure the timely and robust myelination of specific CNS axons and circuits.

## STAR★Methods

### Key resources table

**Table:** 

REAGENT or RESOURCE	SOURCE	IDENTIFIER
**Chemicals, peptides, and recombinant proteins**		

Mivacurium chloride	abcam	cat# ab143667
Capsaicin	Sigma-Aldrich	cat# M2028

**Experimental models: Organisms/strains**		

Zebrafish: myrf^UE70^	Madden et al.^37^, this paper	N/A
Zebrafish: Tg(KalTA4u508)^u508Tg^	Antinucci et al.^57^	ZFIN: ZDB-ALT-200519-9
Zebrafish: Tg(elavl3:Gal4VP16)^ue8Tg^	Mensch et al.^17^	ZFIN: ZDB-ALT-170419-1
Zebrafish: Tg(NBT:KalTA4)	this paper	N/A
Zebrafish: Tg(10xUAS:Sy4xpHy; cryaa:mCherry)	this paper	N/A
Zebrafish: Tg(10xUAS:jGCaMP7s; cryaa:mCherry)	this paper	N/A
Zebrafish: Tg(10xUAS:EGFP-cntn1a)	this paper	N/A
Zebrafish: Tg(10xUAS:TrpV1-tagRFPt)	this paper	N/A
Zebrafish: Tg(10xUAS:axon-GCAMP7s; cryaa:mCherry)	this paper	N/A

**Oligonucleotides**		

Primer sequences indicated in Table S1	this paper	N/A

**Recombinant DNA**		

pTol2- 10xUAS:SypHy	this paper	N/A
pTol2- SypHy–UAS–tdTomato-cntn1a	this paper	N/A
pTol2- SypHy–UAS–BoNTB-mCherry	this paper	N/A
pTol2- SypHy–UAS–vglut1a-mCherry	this paper	N/A
pTol2- SypHy–UAS–nfasca-mCherry	this paper	N/A
pTol2- SypHy–UAS–TRPV1-tagRFPt	this paper	N/A
pTol2- 10xUAS:axon-jGCaMP7s	this paper	N/A
pTol2- 10xUAS: EGFP-2A-tdTomato-cntn1a	this paper	N/A
pTol2- 10xUAS: BoNTB-EGFP-2A-tdTomato-cntn1a	this paper	N/A
tol2kit	Kwan et al.^58^	http://tol2kit.genetics.utah.edu/index.php/Main_Page
Further plasmid DNA for transgenic line generation and further details in STAR Methods	N/A	N/A

**Software and algorithms**		

GraphPad Prism	GraphPad Software	RRID: SCR_015807
Fiji	Schindelin et al.^59^	RRID: SCR_002285
Adobe Illustrator	Adobe	RRID: SCR_010279

### Resource availability

#### Lead contact

Further information and requests for resources and reagents should be directed to and will be fulfilled by the Lead Contact, David Lyons (david.lyons@ed.ac.uk).

#### Materials availability

Reagents generated in this study are available upon request directed to the Lead Contact.

#### Data and code availability

• Microscopy data reported in this paper will be shared by the lead contact upon request.
• This paper does not report original code.
• Any additional information required to reanalyze the data reported in this work paper is available from the Lead Contact upon request.

### Experimental model and subject details

#### Zebrafish Lines and Maintenance

All zebrafish were maintained under standard conditions^60^^,^^61^ in the Queen’s Medical Research Institute BVS Aquatics facility at the University of Edinburgh. Studies were carried out with approval from the UK Home Office and according to its regulations, under project licenses 60/8436, 70/8436 and PP5258250. Adult animals were kept in a 14 hours light and 10 hours dark cycle. Embryos were kept at 28.5°C in 10mM HEPES-buffered E3 Embryo medium or conditioned aquarium water with methylene blue. Embryos were staged according to Kimmel et al.,^62^ and analyzed between 2-7dpf, before the onset of sexual differentiation. The following existing transgenic line was used to drive expression in reticulospinal neurons: Tg(KalTA4u508)^u508Tg^.^60^ The existing pan-neuronal driver line Tg(elavl3:Gal4VP16)^17^ and a novel pan-neuronal line Tg(NBT:KalTA4) (described below) were also used to drive expression in Rohon-Beard and COPA neurons. The recently generated *myrf*^UE70^ mutant line^37^ was used to disrupt myelination and is described further below. The following lines were generated in this study: Tg(NBT:KalTA4); Tg(10xUAS:Sy4xpHy; cryaa:mCherry); Tg(10xUAS:jGCaMP7s; cryaa:mCherry); Tg(10xUAS:EGFP-cntn1a); Tg(10xUAS:TrpV1-tagRFPt); Tg(10xUAS:axon-GCAMP7s; cryaa:mCherry). Throughout the text and figures, ‘Tg’ denotes a stable, germline-inserted transgenic line; and ‘SypHy’ denotes the variant containing four pHluorin molecules, also called ‘Sy4xpHy’.^24^

### Method details

#### myrf^UE70^ generation, genotyping and analysis

The *myrf*^UE70^ allele was generated by injection of Cas9 mRNA and a sgRNA targeting the second exon of *myrf* (target sequence: CATTGACACCAGTATCCTGG) into fertilized wild-type embryos at one-cell stage. Potential founders were grown to adulthood and the UE70 allele isolated from their offspring. UE70 consists of an indel (ΔCC+A) that disrupts the reading frame and introduces a premature stop codon. Homozygous *myrf*^UE70^ mutants have reduced numbers of oligodendrocytes and exhibit hypomyelination of the spinal cord; an in-depth description of this mutant line is published elsewhere.^37^ For SypHy analysis in *myrf*^*UE70*^ mutants, *myrf* ^*UE70*^ heterozygous parents carrying the Tg(KalTA4u508) neuronal driver were in-crossed, and offspring were injected at one-cell stage with SypHy-UAS-tdTomato-cntn1a and *tol2* mRNA, as detailed below. Following SypHy imaging at 4-5dpf, individual larvae were genotyped using primers Myrf F and Myrf R (Table S1) followed by digestion of the PCR product with restriction enzyme PspGI, which cleaves the wild-type PCR product into 131bp and 157bp-long fragments, but not the mutant product, since the *myrf*^UE70^ allele consists of a frameshifting indel which abolishes the PspGI site. Subsequent analysis was performed blinded to genotype.

#### Generation of Tol2kit-compatible entry vectors

To generate tol2kit^58^-compatible entry vectors, we amplified the relevant coding sequences by PCR with Phusion and primers as indicated below and in Table S1, and recombined 25fmol of purified PCR product with 75ng of pDONR221, pDONRP4P1R or pDONRP2RP3 using BP Clonase II.

pME-SypHy was amplified from pcDNA3-SypHluorin 4x^24^ (Addgene #37005) with primers attB1-SypHy and attB2R-SypHy. p5E-SypHy: amplified from UAS:SypHy (this study) with primers attB4-polyA and attB1R-SypHy. p3E-BoNTBmCherry: amplified from UAS:BoNTB-mCherry^31^ with primers attB2-BoNTB and attB3R-polyA. p3E-nfasca-mCherry: amplified from pBH-UAS-HA-NF186-mCherry (this study) with primers attB2-nfasca and attB3R-polyA. p3E-TrpV1RFP: amplified from UAS:TrpV1-tagRFPt (this study) with primers attB2-TRPV1 and attB3R-polyA. pME-TrpV1-RFP islet1: amplified from GAL4VP16,4xUAS:TRPV1-RFPT^42^ with primers attB1_TRPV1_fwd and attB2_TRPV1_rev. pME-jGCaMP7s: amplified from pGP-CMV-jGCaMP7s^63^ (Addgene #104463) with primers attB1-jGCaMP7 and attB2R-jGCaMP7. p5E-axonjGCaMP7s: amplified from pME-axon-jGCaMP7s (this study) with primers attB4-polyA and attB1R-GAP43.

Additional/alternative construction details are as follows:

p3E-(zf)tdTomato-cntn1a: the tagRFPt coding sequence in p3E-tagRFPt-cntn1a^33^ was replaced with a zebrafish codon-optimized non-repetitive sequence of tdTomato amplified from a gBlock (IDT DNA, full sequence available upon request) with primers zftdTomato fwd and zftdTomato rev; and ligated to the p3E-tagRFPtcntn1a backbone amplified with primers zfcntn1a signal rev and zfcntn1a Fwd Phos using T4 DNA ligase.

pME-axonGCaMP7s: pME-jGCaMP7s was digested at the start codon with NcoI (GCCACC*ATG*G, NcoI sequence underlined) into which we ligated two annealed primers, GAP43-fwd and GAP43-rev, encoding the di-palmitoylation motif of murine GAP-43 flanked by overhanging NcoI-compatible ends. This enriches jGCaMP7s in axons compared to non-specific membrane tethers.^43^

p3E-nfasca-mCherry: zebrafish *nfasca* transcripts were amplified by RT-PCR from total brain RNA using primers EcoRI-Kozac-nfasca-F and nfasca-NotI-R (based on cDNA clone IMAGE: 3817913, accession CU458816), cloned into pCRII-TOPO and sequenced. A zebrafish isoform that contains a mucin-like domain (accession FJ669144) was considered as the neuronal form of *nfasca*, similar to the mammalian neuronal isoform NF186. This zebrafish NF186-like cDNA cloned into expression plasmid pUAS-NF186. The HA tag (YPYDVPDYA) was inserted between amino acids 35 and 36 following a predicted secretion signal sequence (SignalP) by recombinant PCR using equimolar amounts of a PCR product containing *nfasca* coding nucleotides 1-105 and the HA tag (amplified with primers EcoRI-Kozac-nfasca-F and nfasca-HA-R) and a PCR product containing nucleotides 106-767 (amplified with primers nfasca-HA-F and nfasca-AgeI-R), and primers EcoRI-Kozac-nfasca-F and nfasca-AgeI-R. The resulting PCR product was cloned into pCRII-TOPO and then used to replace the 5′ end of NF186 in pUAS-NF186 using an EcoRI site upstream of the start codon and an AgeI site downstream of the start codon. The resulting full-length HA-NF-186 fusion was then cloned into a modified version of the Tol2 transgenesis vector pBH-UAS (Michael Nonet, University of Washington) yielding pBH-UAS-HA-NF186. The mCherry coding sequence was then PCR-amplified with primers SacI-nfasca-mCherry-F and mCherry-NotI-R, cloned into pCRII-TOPO, and then used to join the HA-NF186 fusion with the mCherry fusion by ligating a BsiWI/SacI NF186-like cDNA fragment, a SacI/NotI mCherry fragment, and a BsIWI/NotI pBH-UAS-HA-NF186 fragment. This yielded the expression plasmid pBH-UAS-HA-NF186-mCherry, from which the HA-NF186-mCherry coding sequence was amplified to make p3E-nfasca-mCherry using primers attB2-nfasca and attB3R-mCherry.

p3E-vglut1a-mCherry: the coding sequence for zebrafish ortholog *slc17a7a* (vglut1a) was first amplified from a pool of total cDNA from 5dpf wild-type zebrafish from the AB strain using primers slc17a7a F and slc17a7a R, and TOPO-cloned to generate pCRII-slc17a7a. We then amplified this *vglut1a* cDNA with primers attB2-vglut1a and vglut1a-mCherry rev, and the mCherry cDNA from pBH-UAS-HA-NF186-mCherry with primers vglut1a-mCherry fwd and attB3R-polyA. Primers vglut1a-mCherry rev and vglut1a-mCherry fwd exclude the vglut1a STOP codon and add a GGGGS linker between vglut1a and mCherry that provide an overlapping region for recombinant PCR. We combined equimolar amounts of primary PCR products and used recombinant PCR with Phusion to amplify the full-length attB2-vglut1a-mCherry-polyA-attB3R product with the attB-containing primers. We then used BP clonase to recombine this with pDONR-P2rP3 (tol2kit).

pME-EGFP-2A-tdTomato-cntn1a and pME-BoNTB-EGFP-2A-tdTomato-cntn1a: we Phusion-amplified primary PCR product attB1-EGFP-2A with primers attB1-EGFP fwd and 2A-EGFP rev from template plasmid pCS2+EGFP; attB1-BoNTB-EGFP-2A with primers attB1-BoNTB and 2A-EGFP rev from template plasmid UAS:BoTxBLC-GFP;^31^ and 2A-tdTomato-cntn1a-attB2R with primers 2A-(ss)cntn1a fwd and attB2R-cntn1a. We then combined equimolar amounts of primary PCR products as appropriate and used the attB-containing primers in recombinant PCRs to amplify the full-length products, and used BP clonase to recombine these with pDONR221 (tol2kit).

Coding sequences in all 5′-entry vectors are in the reverse orientation and contain a polyadenylation signal, to be compatible with a ‘Janus’ configuration^64^ following LR recombination with a middle-entry vector containing (palindromic) UAS flanked by two minimal promoters and a 3′-entry vector containing another coding sequence in the forward orientation. The sequences of all entry vectors were verified by Sanger sequencing.

#### Generation of Tol2 expression/transgenesis constructs

To generate final Tol2 expression vectors, 10fmol of each entry vector as indicated below and 20fmol of destination vector pDestTol2pA2 from the tol2kit or pDestTol2pA2-cryaa:mCherry^65^ (Addgene #64023) were recombined in a LR reaction using LR Clonase II Plus. 3-4 clones were tested for correct recombination by digestion with restriction enzymes.

10xUAS:SypHy was generated by recombining p5E-10xUAS (tol2kit), pME-SypHy and p3E-polyA(tol2kit). SypHy-UAS-BoNTBmCherry: generated by recombining p5E-SypHy, pME-UAS(Janus)^33^ and p3E-BoNTBmCherry. SypHy-UAS-tdTcntn1a: generated by recombining p5E-SypHy, pME-UAS(Janus), p3E-zftdTomato-cntn1a. 10xUAS:axon-jGCaMP7s: generated by recombining p5E-10xUAS(tol2kit), pME-axonjGCaMP7s, p3E-polyA(tol2kit). axonjGCaMP7s-UAS-TRPV1RFP: generated by recombining p5E-axonjGCaMP7s, pME-UAS(Janus), p3E-TrpV1RFP.

SypHy-UAS-TrpV1RFP: generated by recombining p5E-SypHy, pME-UAS(Janus), p3E-TrpV1RFP. 10xUAS:TrpV1-tagRFPt: generated by recombining p5E-10xUAS(tol2kit), pME-TrpV1-RFP, p3E-polyA(tol2kit). SypHy-UAS-nfascamCherry: generated by recombining p5E-SypHy, pME-UAS(Janus), p3E-nfasca-mCherry. SypHy-UAS-vglut1a-mCherry: generated by recombining p5E-SypHy, pME-UAS(Janus), p3E-vglut1a-mCherry. NBT:KalTA4: generated by recombining p5E-NBT (gift of Dirk Sieger), pME-KalTA4GI,^66^ p3E-polyA(tol2kit). 10xUAS:EGFP-2A-tdtomato-cntn1a: generated by recombining p5E-10xUAS (tol2kit), pME-EGFP-2A-tdTomato-cntn1a, p3E-polyA(tol2kit). 10xUAS:BoNTB-EGFP-2A-tdtomato-cntn1a: generated by recombining p5E-10xUAS (tol2kit), pME-BoNTB-EGFP-2A-tdTomato-cntn1a, p3E-polyA(tol2kit).

#### Generation of transgenic lines

Transgenic lines were generated by injecting 5-10pg of the appropriate plasmid DNA with 25-50pg *tol2* transposase mRNA into wild-type zebrafish eggs at the one-cell stage to promote transgenesis. The plasmid used to make the Tg(UAS:EGFP-cntn1a) has been previously described.^20^ Founder animals were identified by screening F1 offspring for transgenesis markers, and F1 offspring were raised to generate stable transgenic lines.

#### Single cell labeling

One-cell stage Tg(KalTA4u508), Tg(elavl3:Gal4VP16) or Tg(NBT:KalTA4) (neural beta-tubulin promoter) eggs were injected with 1-5pg of plasmid DNA containing UAS-driven SypHy or other reporters as indicated with 25pg of *tol2* transposase mRNA, to mosaically express axonal reporters in neurons. For some experiments single-neuron labeling was also obtained by crossing Tg(KalTA4u508) with Tg(UAS:SypHy) transgenic fish. For certain experiments neuronal driver lines were maintained in a *myrf*^UE70^ heterozygous background, or with in a Tg(UAS:TRPV1-RFP) background.

#### Live-imaging

The workflow for imaging zebrafish larva is detailed in Williamson et al.^67^ Briefly, at 3-5dpf, larvae were paralysed by a 5-minute bath-application of 1.5mg/ml mivacurium chloride (Abcam) in E3 embryo medium and immobilised in 1.5% low melting-point agarose on their sides. Animals were imaged using a Zeiss LSM880 confocal with Airyscan in Fast mode and a Zeiss W Plan-Apochromat 20x/1.0 NA water-dipping objective, and a 488nm (SypHy), 568nm (tdTomato, tagRFPt) and 594nm (mCherry) laser. We used 3-4.5X zoom and acquired 1500-2300 (X) x 50-500 (Y) pixels, sampling 100-140μm of axonal length (∼16 pixels/μm), over a small z stack (4-15 z-slices, 1-5μm z-step) that sampled the depth of the axon and collateral branches. This was acquired repeatedly at a frequency of 0.6-2.3 Hz (mean 1.1Hz), for 5-20 minutes. For experiments in which axons co-expressed a static red fluorescent marker (e.g., BoNTB-mCherry, tdTomato-cntn1a, TrpV1-tagRFPt), the red channel was acquired once, in an optimally-sectioned z stack, in the same region as SypHy, with higher pixel dwell time to reduce noise. For imaging SypHy with a co-expressed dynamic red marker (e.g., nfasca-mCherry, vglut1a-mCherry), we acquired every line of each channel sequentially to maximize temporal resolution. For axonal Ca^2+^ imaging, a higher resolution image of TRPV1-RFP and axon-jGCaMP7 expression was acquired, then a 3-5 minute axon-jGCaMP7 timelapse was acquired at ∼1Hz before and 15 minutes after vehicle or capsaicin treatment (detailed below).

#### Chemogenetic stimulation

Capsaicin (Sigma-Aldrich) was prepared as a 5mM primary stock in 100% DMSO and stored at −80°C. For chronic treatments, the transgenic lines Tg(UAS:TRPV1-RFP) and Tg(UAS:GFP-Cntn1a) were crossed into the Tg(KalTA4u508) background to drive mosaic expression of both transgenes in reticulospinal neurons. Animals with individual reticulospinal axons expressing both transgenes were live-imaged at 3dpf as above and then returned to E3 embryo medium. Animals were treated with either vehicle (1% DMSO) or capsaicin (1μM capsaicin in 1% DMSO) in E3 embryo medium for 4 hours after imaging at 3dpf, and then every day until 7dpf. Animals were re-imaged as above at 5dpf to obtain time-course data, and were kept individually in 12-well plates in-between imaging sessions. For acute capsaicin treatment of axon-GCaMP7s and SypHy expressing larva, before imaging as detailed above, a small window of agarose was removed along the trunk of the animal to facilitate diffusion of 1μM capsaicin-containing (or vehicle) E3 embryo medium, which was allowed to equilibrate for 10 minutes before re-imaging.

#### Image processing and analysis

We used Fiji^59^/ImageJ and Python scripts for most image processing and analysis, and Adobe Illustrator for figure panels, using average or maximum-intensity projections and cropped representative x-y areas. SypHy time-lapses were pre-processed by bleach-correction with exponential curve fitting, and registration, where needed, using the template-matching plugin or custom written ImageJ macros that apply rigid transformation. Putative SypHy events were identified in the pre-processed (but otherwise raw) timelapse, aligned to a ΔF/F_avg_ timelapse (proportional increase over the all-time average intensity) to aid discrimination of the event start. For each potential event, we defined its region of interest using Fiji’s Wand tool to select the maximum intensity pixel and connected region over a third of the maximum fluorescence intensity. We used this ROI to determine the increase in fluorescence intensity relative to the baseline, defined as the average intensity in the ten frames preceding the event (F_0_), and only considered events with an increase that was 5-fold greater than the baseline standard deviation, present in at least 4 frames (∼4 s). The amplitude was defined as the highest proportional increase that the ROI reached over the baseline (ΔF/F_0_) during the peak period, defined as the first ten frames. Duration was defined as the time until fluorescence decreased to within one standard deviation of the baseline. We also calculated the cumulative displacement of the event using the maximum intensity pixel and, where substantial (> ∼2μm) classified this displacement as unidirectional or bidirectional for axonal events, and anterograde or retrograde (away or toward the axon, respectively) for collateral events. We excluded from further analysis events that showed motion throughout their duration or > 10μm displacement, but included those events that showed some, limited displacement (< 10μm), and that were static for longer periods than they were moving (these could represent, for example, instances of kiss-and-run exocytosis). Collection of these parameters was automated using custom written ImageJ macros, but all events were manually inspected and parameters corrected where needed. SypHy event frequency was normalized to axonal or collateral length, which were measured in an average-intensity projection of all time frames; and normalized to imaging duration. Kymographs were made using the Fiji Multi Kymograph plugin.

For analysis of tdTomato-cntn1a expressing axons, myelin sheath location and lengths were inferred from tdTomato-cntn1a negative gaps that bridged the axon’s thickness, as before.^20^ The percentage of myelination was calculated as the summed length of tdTomato-negative gaps in the axon, including those that are incomplete at the edges of the field of view, divided by the total length of axon sampled in the field of view. We considered axons yet-to-be-myelinated if they had < 5% myelination, and axons actively undergoing myelination if they had > 5% myelination or > 2 putative heminodes. All images were taken at the mid-trunk level, approximately between somites 10-20. We classified the location of all axonal SypHy events as tdTomato^+^ or tdTomato^-^, and further classified tdTomato^+^ Syphy events as ‘heminodal’ when occurring at putative heminodal locations, defined as the first 3μm of tdTomato^+^ axon bordering a tdTomato^-^ gap. Collaterals branching points were always located in non-myelinated tdTomato^+^ parts of the axon. We normalized SypHy frequencies to tdTomato^+^ length and tdTomato^-^ length where appropriate. To calculate the predicted heminodal SypHy frequency in each axon, we scaled the overall SypHy frequency in the tdTomato^+^ part of the axon to a 3μm window (expressed as number of events/3μm/h), which would reflect a uniform distribution, and compared this to the observed overall heminodal frequency divided by the number of heminodes. For sheath-centric analyses, we only considered complete gaps, i.e., excluding incomplete gaps at the edge of the field of view. We considered an heminode as a node if its length was < 1μm and immediately flanked by another gap. For myelin growth analysis, we identified individual sheaths over time based on axonal landmarks, including collateral branches, which have unique morphologies. We considered sheaths ‘free’ to grow if at least one of their sides was not bordered by nodes or collaterals. For correlations between sheath growth and SypHy, we considered only sheaths free to grow in our analyses.

For Rohon-Beard SypHy analyses, individual neurons were identified by their large soma, very dorsal location in the spinal cord, and stereotyped longitudinal large-calibre branches emerging from the soma, with ramified branches that exit the spinal cord and innervate the periphery. SypHy imaging was performed as for reticulospinal neurons, focusing on the anterior branch projecting toward the postsynaptic targets (the ‘axonal’ branch). For COPA SypHy analyses, individual neurons were identified by their unique triangular soma morphology and ascending commissural morphology. SypHy imaging was performed in the axon after it crosses to the contralateral spinal cord and where it joins the dorsal longitudinal fasciculus, within 2-3 somites of the soma.

For vglut1a-mCherry co-localization analyses along axons, we used two-channel kymographs to aid identification of static and dynamic mCherry^+^ puncta (e.g., Figure S1), and then determined if SypHy activity occurred at any point during imaging in identified static mCherry^+^ puncta, starting from a location in which there was a dynamic mCherry^+^ punctum, or if trafficking SypHy^+^ particles co-localized with dynamic mCherry^+^ puncta.

For axon-GCaMP7s Ca^2+^ analysis, a well-defined collateral branching from the main axon was defined as a ROI and background-subtracted proportional change over the average was calculated ΔF/F_avg_. The pre-treatment timelapse was considered a measure of baseline neuronal activity, and for the post-treatment timelapse, the proportional change in fluorescence ΔF/F_0_ was calculated using the average of pre-treatment timelapse as F_0_. For analysis of individual axon myelination during chronic stimulation, one axon was analyzed per animal, and sheath length inferred from FP-cntn1a gaps measured using Fiji as before.^20^

### Quantification and statistical analysis

All graphs and statistical tests were carried out using GraphPad Prism. All data were averaged per biological replicate (N represents number of animals), except where otherwise noted in the figure legends. Data were tested for normal distribution using D’Agostino & Pearson and Shapiro-Wilk normality tests. Normally distributed groups were compared using two-tailed unpaired Student’s t test or one-way ANOVA with correction for multiple comparisons, and non-normally distributed groups were compared using the Mann-Whitney U test, Wilcoxon matched-pairs signed rank test for paired data, or Kruskal-Wallis test with Dunn’s correction for multiple comparisons, as indicated in figure legends. For correlation analysis, we used parametric Pearson’s test for linear correlation as indicated in Figure legends. We considered a difference significant when p < 0.05, and indicate p values in figure legends and in figures as follows: no indication or ‘ns’ p > 0.05, ^∗^p < 0.05, ^∗∗^p < 0.01, ^∗∗∗^p < 0.001. Error bars illustrate mean ± standard deviation for normally distributed data or median and interquartile range for non-normally distributed data as indicated in figure legends.

## Supplemental information

Document S1. Figures S1–S4 and Table S1

Document S2. Article plus supplemental information
