# Supplementary material for: Myelination induces axonal hotspots of synaptic vesicle fusion that promote sheath growth
Source: Curr Biol. 2021 Sep 13;31(17):3743–3754.e5. doi: 10.1016/j.cub.2021.06.036 (PMC8445327; doi:10.1016/j.cub.2021.06.036)
Supplement: Document S1. Figures S1–S4 and Table S1 [file mmc1.pdf]

Current Biology, Volume *31*

Supplemental Information

Myelination induces axonal hotspots of synaptic vesicle fusion that promote sheath growth

Rafael G. Almeida, Jill M. Williamson, Megan E. Madden, Jason J. Early, Matthew G. Voas, William S. Talbot, Isaac H. Bianco, and David A. Lyons

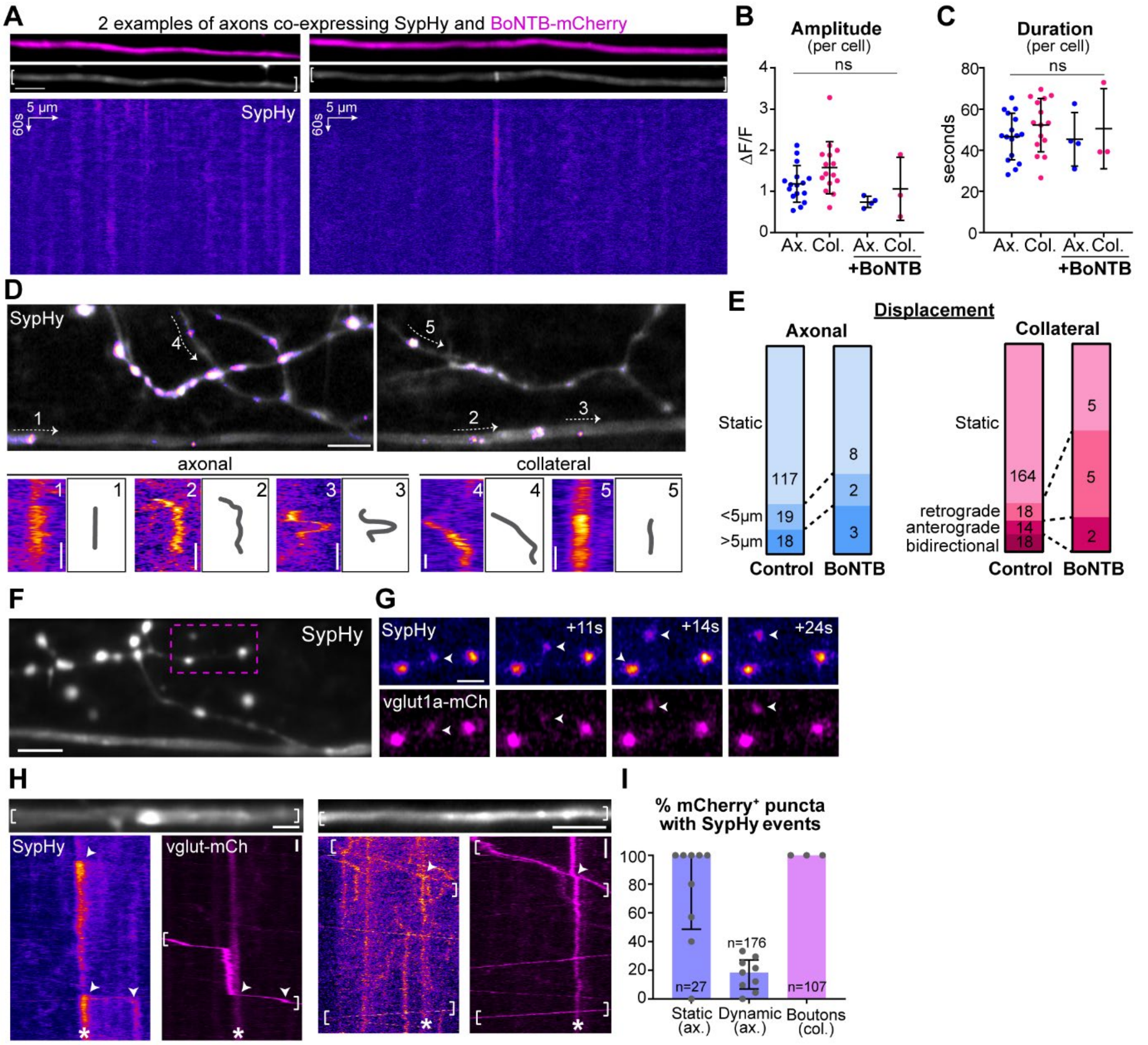

Figure S1- Further SypHy characterization, related to Figure 1.

A. Two examples of reticulospinal axons co-expressing SypHy and BoNTB-mCherry, which silences SypHy activity.
B-C. SypHy event amplitude and duration per cell; note trend to amplitude reduction in remaining events in BoNTB$^+$ axons. (B and C: all comparisons p>0.05, ANOVA with Bonferroni's multiple comparisons correction. 16 control axons from 16 animals; 4 BoNTB axons from 4 animals, averaged per animal).
D-E. Kymographs of static and slightly displaced axonal and collateral events and quantification (binned into <5µm or >5µm cumulative displacement, retrograde, anterograde or bidirectional). Note proportional increase of dynamic events in silenced axons.
F-H. Examples of SypHy activity and concomitant vglut1a-mCherry vesicle trafficking in reticulospinal collaterals (G) and axons (H). Note SypHy events (e.g. arrowheads in H) and dynamic SypHy$^+$ puncta (brackets in H) co-localize with vglut1a-mCherry$^+$ puncta (asterisks) or trafficking particles (bracketed).
I. Quantification of SypHy and vglut1a-mCherry overlap as proportion of mCherry$^+$ puncta that co-localize with SypHy events in the imaging period. mCherry$^+$ static axonal puncta and boutons in collateral branches co-localize with SypHy, as do a proportion of dynamic axonal puncta (n indicates mCherry$^+$ axonal puncta from N=9 axons in 5 fish, and collateral puncta are in 3 axons in 3 animals).
Scale bars: 5µm (A-F), 2µm(G-H), 60s (A-H). Graphs display mean and standard deviation.

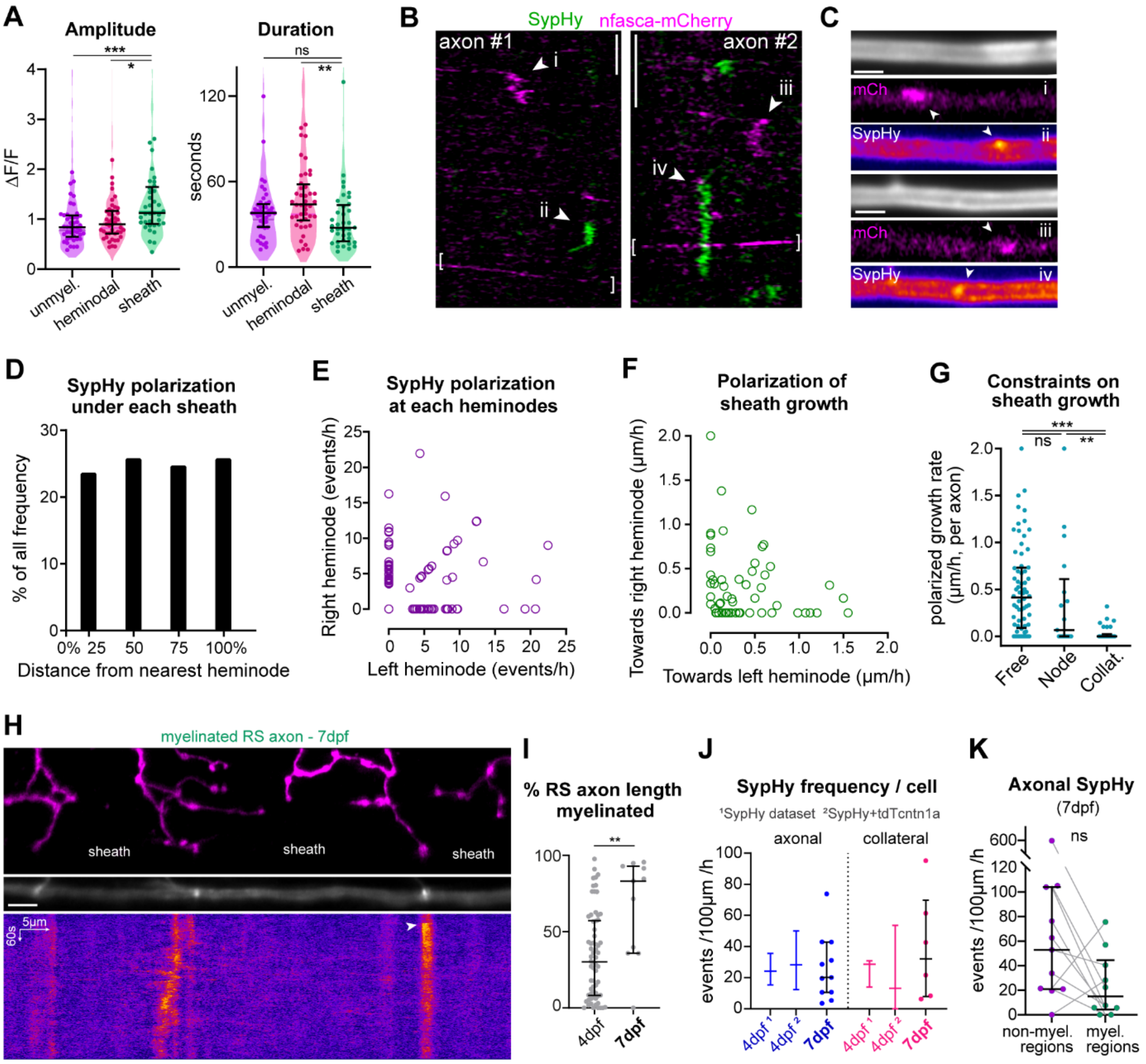

Figure S2- Further SypHy-myelination characterization, related to Figures 2, 4-5.

A. SypHy amplitude and duration in reticulospinal axons (amplitude: $p<0.001$ non-myelin. vs sheath, $p=0.012$ hemi vs sheath, $p>0.05$ non-myelin. vs hemi; duration: $p=0.003$ hemi vs sheath, other comparisons $p>0.05$; Kruskal-Wallis with Dunn's multiple comparisons test, N=37-45 axons).
B-C. Kymographs of two examples of SypHy and nfasca-mCherry trafficking (no co-localization). Frames corresponding to events i-iv shown in C.
D. SypHy polarization under each sheath (distance normalized to nearest heminode as 0% and to sheath centre as 100%). 50 sheaths, 35 axons, 33 animals.
E. Most sheaths show polarized heminodal SypHy activity. 161 sheaths, 60 axons, 52 animals.
F. Most sheaths grow asymmetrically towards one heminode. 66 sheaths, 32 axons, 30 animals.
G. Sheaths not flanked by nodes or collaterals grow faster ($p=0.213$ free vs node, $p<0.001$ free vs collateral, $p=0.039$ node vs collateral, Kruskal-Wallis with Dunn's multiple comparison test; 68 free heminodes, 17 node-flanked heminodes and 30 collateral-flanked heminodes, from 11-29 axons).
H-I. Increased myelination of reticulospinal axons at 7dpf ($p=0.005$, Mann-Whitney test, 66 RS axons in 57 animals at 4dpf and 11 axons from 9 animals at 7dpf).
J. Axonal and collateral SypHy frequency at 4 and 7dpf (axon: all comparisons $p>0.05$; collat.: all comparisons $p>0.05$; Kruskal-Wallis with Dunn's multiple comparison test; N as in Fig 1, 2 and S2I).
K. SypHy frequency in myelinated regions of 7dpf reticulospinal axons tends to be lower than at non-myelinated regions, similar to 4dpf ($p=0.065$, Wilcoxon matched-pairs signed rank test).

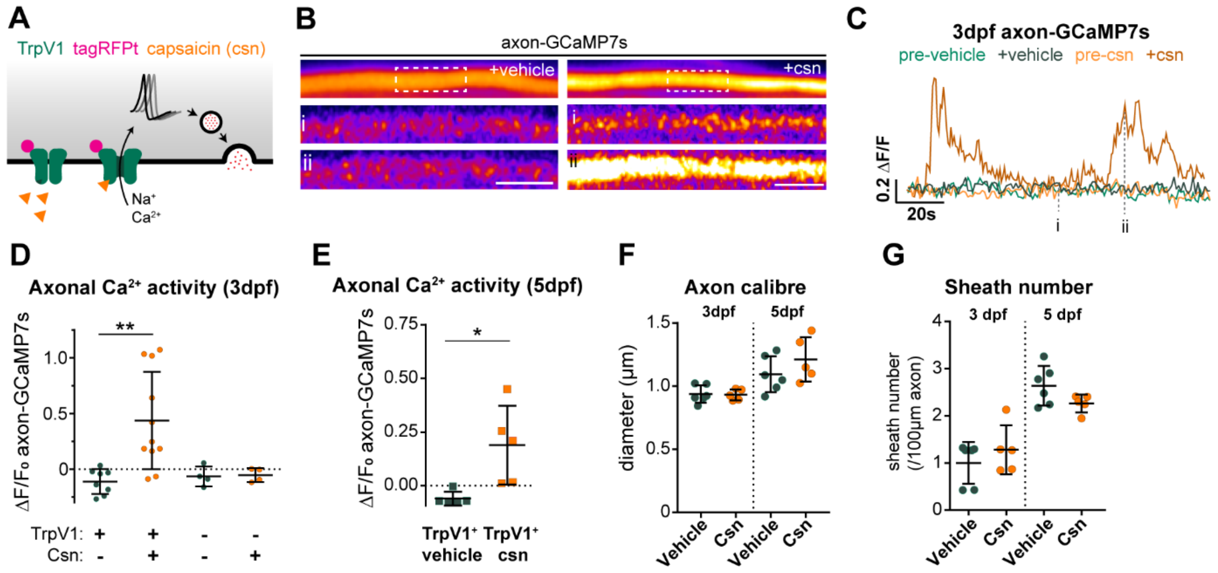

Figure S3- Further chemogenetic stimulation characterization, related to Figure 6.

A. Schematic depicting TrpV1 chemogenetic stimulation approach to increase vesicular fusion.
B. Axon-GCaMP7s in 3dpf TrpV1^{+} axons, top panels are average projection, panels i and ii below are individual frames of the timepoints indicated in C. Only csn increases Ca^{2+} activity.
C. Fluorescence time-course of boxed regions in B. Frames corresponding to timepoints i and ii displayed in B.
D-E. Quantification of average axonGCaMP7s activity at 3dpf (D) and 5dpf (E), showing specific increase in csn-treated TrpV1^{+} axons (3dpf: csn vs vehicle treatment of TrpV1^{+} axons: p=0.003, Student's t-test, 19 TrpV1^{+} axons in 19 animals, 8 TrpV1^{-} axons in 8 animals; 5dpf: p=0.017, 10 TrpV1^{+} axons in 10 animals).
F. Reticulospinal axon calibre in control and stimulated axons (p>0.05 for vehicle vs csn comparisons, ANOVA with Bonferroni's multiple comparison test, n-5-6 axons from 5-6 animals).
G. Sheath number in control and stimulated axons (p>0.05 for vehicle vs csn comparisons, ANOVA with Bonferroni's multiple comparison test, n-5-6 axons from 5-6 animals).
Scale bars: 2µm (B). Graphs display mean and standard deviation.

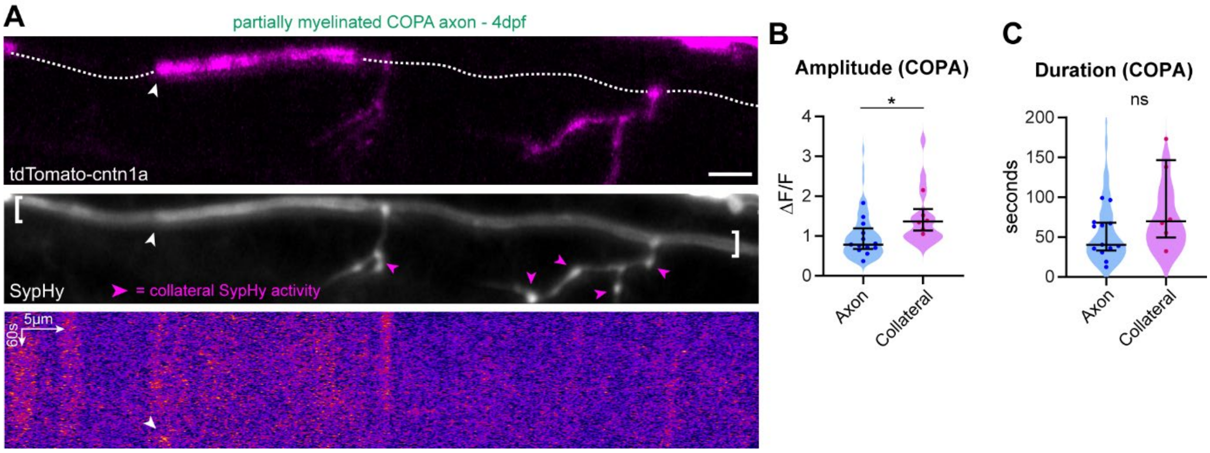

Figure S4- Further SypHy characterization in COPA neurons, related to Figure 7.

A. Example of SypHy activity along a partially myelinated COPA axon. Kymograph representative of bracketed axonal region.
B-C. Quantification of SypHy event amplitude and duration in COPA axons and collaterals (B: p=0.017, C: p=0.127, Mann-Whitney tests, 13 axons and collateral branches from 6 neurons).
Scale bars: 5µm (A). Graphs display median and interquartile range.

Primer	Sequence (5’-3’)
attB1-SypHy	GGGGACAAGTTTGTACAAAAAAGCAGGCTGCCACCATGGACGTGGTGA
attB2R-SypHy	GGGGACCACTTTGTACAAGAAAGCTGGGTTTACTGATTGGAGAAGGAGGTGGGCGCAC
attB4-polyA	GGGGACAACTTTGTATAGAAAAGTTGAAAAAACCTCCCACACCTCCCCC
attB1R-SypHy	GGGGACTGCTTTTTTGTACAAACTTGGCCACCATGGACGTGGTGAATCAGCTGG
attB2-TRPV1	GGGGACAGCTTTCTTGTACAAAGTGGGCCACCATGGAACAACGGGCTAGC
attB3R-polyA	GGGGACAACTTTGTATAATAAAGTTGAAAAAACCTCCCACACCTCCCCCTG
attB1-jGCaMP7	GGGGACAAGTTTGTACAAAAAAGCAGGCTGCCACCATGGTCGACTCATCACGTC
attB2R-jGCaMP7	GGGGACCACTTTGTACAAGAAAGCTGGGTTTTACTTCGCTGTCATCATTTGTACAAAC
attB1R-GAP43	GGGGACTGCTTTTTTGTACAAACTTGGCCACCATGCTGTGCTGTATGAGAA
GAP43-fwd	CATGCTGTGCTGTATGAGAAGAACCAAACAGGTTGAAAAGAATGATGAGGACCAAAAGATTGGTAG
GAP43-rev	CATGCTACCAATCTTTTGGTCCTCATCATTCTTTTCAACCTGTTTGGTTCTTCTCATACAGCACAG
zftdTomato fwd	/5Phos/ATGGTTTCAAAAGGCGAAGAAG
zftdTomato rev	/5Phos/CTTGTAAAGCTCATCCATTCCG
zfcntn1a signal Rev	GGTCCCGAAGCCTGCAGACTCACC
zfcntn1a Fwd	CCGGTGTTCGAGGAGCAGCCGC
attB1_TRPV1_fwd	GGGGACAAGTTTGTACAAAAAAGCAGGCTGCCACCATGGAACAACGGGC
attB2R_TRPV1_rev	GGGGACCACTTTGTACAAGAAAGCTGGGTTTAATTGTACAGCTCGTCCATGC
attB2-BoNTB	GGGGACAGCTTTCTTGTACAAAGTGGGCCACCATGCCCGTGACAATTAACA
attB2-nfasca	GGGGACAGCTTTCTTGTACAAAGTGGGCCACCATGTGGACACAGAGGCGGTGGGC
attB3R-mCherry	GGGGACAACTTTGTATAATAAAGTTGTTTACTTGTACAGCTCGTCCATGCC
gap43-fwd	CATGCTGTGCTGTATGAGAAGAACCAAACAGGTTGAAAAGAATGATGAGGACCAAAAGATTGGTAG
gap43-rev	CATGCTACCAATCTTTTGGTCCTCATCATTCTTTTCAACCTGTTTGGTTCTTCTCATACAGCACAG
attB1R-GAP43dipalm	GGGGACTGCTTTTTTGTACAAACTTGGCCACCATGCTGTGCTGTATGAGAA
MyrfF	AACTGTGCGTAGGAACACGATA
MyrfR	TGGACCTCCGTGAAACAACTG
EcoRI-Kozac-nfasca-F	GAATTCCACCATGTGGACACAGAGGCGG
nfasca-NotI-R	GCGGCCGCTATGCCAAAGAGTAGATGGC
nfasca-HA-R	AGCGTAGTCTGGGACGTCGTATGGGTACTGGATCTTTGGGTCCAG
nfasca-HA-F	TACCCATACGACGTCCCAGACTACGCTCAAGAGCTGAAACAGCCC
nfasca-AgeI-R	ATCTTGGTGCTGGACAAG
SacI-nfasca-mCherry-F	GAGCTCAGAAGCCACATCACCTGTCAATGCCATCTACTCTTTGGCAATGGTGAGCAAGGGCGAG
mCherry-NotI-R	GCGGCCGCTACTTGTACAGCTCGTCCATGCC
slc17a7aF	ATGGAGATCCGTCCGGACCGCTTTAAAG
slc17a7aR	CTATGTGAGCTCTCTTTCTCCCTCTCCCCC
attB2-vglut1a	GGGGACAGCTTTCTTGTACAAAGTGGGCCACCATGGAGATCCGTCCGGACC
vglut1a-mCherry rev	CGGACCCTCCGCCTCCTGTGAGCTCTCTTTCTCC
vglut1a-mCherry fwd	GGAGGCGGAGGGTCCGTGAGCAAGGGCGAGG
attB1-EGFP	GGGGACAAGTTTGTACAAAAAAGCAGGCTGCCGCCACCATGGTGAGCAAGGGCGAGGAG
attB1-BoNTB	GGGGACAAGTTTGTACAAAAAAGCAGGCTGCCACCATGCCCGTGACAATTAACA
2A-EGFP rev	GTCTCCTGCTTGCTTTAACAGAGAGAAGTTCGTGGCTCCGGATCCCTTGTACAGCTCGTCCATGCCG
2A-(ss)cntn1a fwd	GCCACGAACTTCTCTCTGTTAAAGCAAGCAGGAGACGTGGAAGAAAACCCCGGTCCTATGATTCCAGAGGCCTTCCAGC
attB2R-cntn1a	GGGGACCACTTTGTACAAGAAAGCTGGGTTCAGAGCATCAGAGTCCAGAGGAGC

Table S1 – Primers used in this study. Related to STAR Methods
